# The presymptomatic treatment with 3HFWC nanosubstance decreased plaque load in 5XFAD mouse model of Alzheimer's disease

**DOI:** 10.1111/cns.14188

**Published:** 2023-03-27

**Authors:** Milka Perovic, Jelena Ciric, Valentina Matovic, Maja Srbovan, Djuro Koruga, Selma Kanazir, Sanja Ivkovic

**Affiliations:** ^1^ Department of Neurobiology, Institute for Biological Research “Sinisa Stankovic” ‐ National Institute of Republic of Serbia University of Belgrade Belgrade Serbia; ^2^ NanoLab, Biomedical Engineering, Faculty of Mechanical Engineering University of Belgrade Belgrade Serbia; ^3^ TFT Nano Center Belgrade Serbia; ^4^ Department of Molecular Biology and Endocrinology, “VINČA” Institute of Nuclear Sciences ‐ National Institute of Republic of Serbia University of Belgrade Belgrade Serbia

**Keywords:** 3HFWC, 5XFAD mice, Alzheimer's disease, cortex, machine learning, near‐infrared spectroscopy, prevention

## Abstract

**Introduction:**

In the present study, we assessed the effects of the hyper‐harmonized‐hydroxylated fullerene–water complex (3HFWC) on Alzheimer's disease (AD) neuropathological hallmarks in 5XFAD mice, an AD animal model.

**Methods:**

The 3‐week‐old 5XFAD mice were exposed to 3HFWC water solution ad libitum for 3 months in the presymptomatic phase of pathology. The functional effects of the treatment were confirmed through near‐infrared spectroscopy (NIRS) analysis through machine learning (ML) using artificial neural networks (ANNs) to classify the control and 3HFWC‐treated brain tissue samples. The effects of 3HFWC treatment on amyloid‐β (Aβ) accumulation, plaque formation, gliosis, and synaptic plasticity in cortical and hippocampal tissue were assessed.

**Results:**

The 3HFWC treatment significantly decreased the amyloid‐β plaque load in specific parts of the cerebral cortex. At the same time, 3HFWC treatment did not induce the activation of glia (astrocytes and microglia) nor did it negatively affect synaptic protein markers (GAP‐43, synaptophysin, and PSD‐95).

**Conclusion:**

The obtained results point to the potential of 3HFWC, when applied in the presymptomatic phase of AD, to interfere with amyloid plaque formation without inducing AD‐related pathological processes such as neuroinflammation, gliosis, and synaptic vulnerability.

## Graphical abstract

A potential of 3HFWC, a hydroxylated fullerenol derivative, to affect amyloid‐b aggregation was evaluated in the 5xFAD transgenic mouse model of Alzheimer's disease (AD). The effect of the treatment was confirmed through NIRS analyses and applied machine learning. Our study showed that the presymptomatic, 3‐month‐long 3HFWC treatment significantly reduced the plaque load in the cortex of treated animals. At the same time, the treatment did not induce astrogliosis, microglial activation, and synaptic vilnerability. These data suggest that 3HFWC can be considered an adjuvant in the development of new therapeutic strategies in AD treatment.

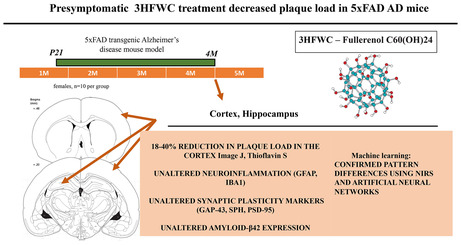

## 1 INTRODUCTION

Alzheimer's disease (AD) is a progressive neurodegenerative disease and the most common cause of dementia in the elderly, characterized by a gradual decline of cognitive abilities accompanied by depression, anxiety, and increased frailty. Ultimately, the patients are stripped from the ability to lead independent lives, creating significant health, economic, and social problems.^1^

Although the etiology of AD is still unknown, the prevalent “amyloid hypothesis” states that the aggregation of amyloid‐β (Aβ) peptide and the formation of senile plaques is responsible for the development of neurodegenerative processes and the consequent loss of memory and cognitive abilities.^2^ The increased production of the main toxic form of Aβ, Aβ_42_,^3^, ^4^ leads to its polymerization and formation of amyloid plaques, induction of inflammation, neurofibrillary tangles formation, synapse dysfunction, and neuronal death, resulting in dementia.^5^, ^6^

The administration of nanoparticles such as carbon nanotubes, fullerenes, and graphene can modulate Aβ aggregation.^7^, ^8^, ^9^, ^10^, ^11^ Fullerenes are particularly interesting as promising therapeutics in biomedical applications due to their small size (approximately 1 nm in diameter), caged structure, and the capacity to cross biological barriers.^12^, ^13^, ^14^ Importantly, fullerene and its derivatives were shown to have anti‐amyloid properties,^9^, ^15^, ^16^, ^17^, ^18^, ^19^ suggesting their usage as promising candidates in the AD treatment. The creation of water‐soluble fullerenols such as harmonized‐hydroxylated fullerene–water complex ‐ 3HFWC (C_60_(OH)_24–45_)^20^, ^21^ largely increased their therapeutic potential. The functional effects of 3HFWC on the CNS were recently confirmed through the changes in the fMRI status of specific brain regions.^22^ Machine learning (ML) through artificial neural network (ANN) training is an additional powerful tool for determining the altered properties of the CNS, that is lately gaining importance and applicability.^23^, ^24^ Using sophisticated algorithms operating on large‐scale, heterogeneous datasets ML uncovers useful patterns that would be difficult or impossible for even well‐trained individuals to identify.^25^ There are already many applications of ML for the detection of altered properties of the CNS, including the presence of Alzheimer's disease,^23^, ^24^ indicating the significance of machine learning application in biomedicine.

The effects of fullerenes or its derivatives in animal models of Alzheimer's disease have not been analyzed so far. In the present study, we used a 5XFAD transgenic mouse model of AD^26^ to assess the in vivo effects of the treatment with 3HFWC (the second fullerene derivative) on AD‐related pathology, including amyloid deposition, inflammation, and synaptic changes. Given the gender prevalence in AD (twice as high in the female population), only female mice were used herein. The 3‐month‐long 3HFWC treatment was applied immediately after weaning (3‐week‐old mice) at the presymptomatic phase of the disease. The ML‐confirmed effects of 3HFWC treatment were further examined on gliosis, synaptic protein levels, Aβ accumulation, and the distribution of amyloid plaques.

## 2 MATERIALS AND METHODS

### 2.1 Ethics statement

All animal procedures followed Directive (2010/63/EU) on the protection of animals used for experimental and other scientific purposes and were approved by the Ethical Committee for the Use of Laboratory Animals (resolution No 03–03/19) of the Institute for Biological Research “Sinisa Stankovic,” University of Belgrade. Minimal numbers of animals were used, and all efforts were made to minimize animal suffering.

### 2.2 Experimental animals and 3HFWC treatment

A total of 20 transgenic (Tg) females of 5XFAD mice as an animal model of Alzheimer's disease were used in this study. 5XFAD mice express human amyloid precursor protein (APP) and presenilin 1 (PSEN1) transgenes with a total of five AD‐linked mutations (three in APP and two in PSEN1) under transcriptional control of neuron‐specific murine Thy‐1 promoter.^26^ 5XFAD transgenic male mice were crossed with C57BL/6xSJL female mice (both from Jackson Laboratory), and F1 offspring was used for the analyses. The animals (*n* = 5 per cage) were housed under standard conditions (23 ± 2°C, 60%–70% relative humidity, 12‐h light/dark cycles), and their health status was routinely checked. Commercial rodent chow pellets (Veterinarski zavod Subotica) were available ad libitum (AL).

On postnatal day 21 (P21), females were randomly divided into two groups: the control group (5XFAD‐Ctrl, *n* = 10) and the treated group (5XFAD‐3HFWC, *n* = 10). Treated animals were drinking 3HFWC (hydroxylated fullerene water complex (C_60_(OH)24–45), TFT Nano Center) solution (0.15 g/L in distilled/tap water 3:1) AL, instead of regular water for 3 months. Control groups were drinking distilled/tap water (3:1) AL.

### 2.3 Sample preparation

At the end of the treatment, animals were anesthetized (100 mg/kg, Ketamidor, Richter Pharma; 16 mg/kg Xylased, Bioveta) and transcardially perfused with 0.1 M ice‐cold phosphate‐buffered saline (PBS). The brains were harvested, and cortices and hippocampi were extracted from the right hemisphere, quickly frozen, and stored at −80°C until used for Western blot analyses. For immunohistochemistry and histological staining, left hemispheres were fixed in 4% paraformaldehyde (PFA) in PBS for 24 h and then cryoprotected in graded sucrose solutions (10%–30% w/v sucrose/ PBS), snap‐frozen, and stored at −80°C. Brains were cryocut serially (30 μm thick; Leica) and further stored free‐float in a cryoprotective buffer (0.05 M phosphate buffer, 25% glycerol, and 25% ethylene glycol) at −20°C. The level of sections was approximately −1.656 to −2.255.^27^

### 2.4 The near‐infrared spectroscopy (NIRS) and machine learning

#### 2.4.1 The near‐infrared spectroscopy (NIRS)

The NIR optical absorption spectra have been registered using the Lambda 950 (Perkin Elmer) spectrometer, equipped with a standard tungsten halogen lamp and (PbS) detector. The wavelength region of interest was 250–3000 nm, and the resolution was set to 4 nm. The instrument was connected to a PC with the Windows 7 operating system and controlled by Perkin Elmer UV WIN LAB Explorer.

#### 2.4.2 Machine learning

Machine Learning (ML) using artificial neural networks (ANNs) was applied in order to classify the control and 3HFWC‐treated brain tissue samples. This classifier method was used on the same dataset and evaluated by Area Under the Curve (AUC).^28^ Brain sections from control and treated animals (*n* = 10 per each group), at the levels of putamen (+0.74 to the bregma, anterior, A) and dorsal hippocampus (−2.06 to the bregma, posterior, P)^27^ were presented in the form of a binomial variable, where 0 indicated control samples and 1 was indicated treatment samples.

Artificial neural networks were trained so that a particular input led to the specific target output. Validation and test data sets are each set to 15% of the original data. With these settings, the input vectors and target vectors were randomly divided into three sets: 70% were used for training, 15% were used to validate that the network is generalizing and to stop training before overfitting, and 15% were used as a completely independent test of network generalization.^29^ The standard network used for pattern recognition is a two‐layer feedforward network, with a sigmoid transfer function in the hidden layer and a transfer function in the output layer. The number of input neurons is “*n*,” and hidden neurons are set to 9 (2n + 1 is a number of input spectrum), according to the Kolmogorov mapping network.^30^, ^31^ The number of output neurons is a set of 2 and equal to the number of elements in the target vector (the number of categories). The classifier method was used on the same dataset with cross‐validation and AUC evaluation, and results were compared between anterior (A) and posterior (P) sections. Spectra were normalized using Standard Normal Variates (SNV).^32^ We chose the classification approach to construct the model with all samples distributed into control (0) and treatment (1). Algorithms were evaluated using Receiver Operating Characteristic (ROC) curve and AUC score. A receiver operating characteristic (ROC) graph is a technique for visualizing, organizing, and selecting classifiers based on their performance, especially useful for data with skewed and unequal class distribution usually found in disease detection. ROC graphs are two‐dimensional graphs in which the true positive rate (sensitivity) is plotted on the *Y*‐axis and the false positive rate (1‐sensitivity) is plotted on the *X*‐axis.^33^ A perfect test would show points in the upper‐left corner, with 100% sensitivity and 100% specificity. The ROC curve coupled with its AUC is a standard method used to estimate the diagnosis potential of a classifier in clinical applications. A larger AUC indicates higher prediction ability.^34^

### 2.5 Western blot analysis

For Western blot analyses, cortices and hippocampi were homogenized in 10 vol (w/v) of RIPA buffer (50 mM Tris‐Cl, pH 7.5, 150 mM NaCl, 1% NP‐40, 0.1% SDS, 10 mM EDTA, pH 8.0, 10 mM EGTA, pH 7.2, 0.5% Triton X‐100) with protease and phosphatase inhibitors (Roche Diagnostics). Protein concentrations were determined using a Micro BCA Protein Assay Kit (Pierce Biotechnology). Proteins (5 μg per lane) were separated on 12% polyacrylamide gels and transferred onto Immobilon‐P membrane (Merck Millipore). After blocking (1 h in 5% non‐fat dry milk dissolved in Tris‐buffered saline/0.1% Tween 20 [TBST]), membranes were incubated overnight at +4°C, with mouse anti‐hAPP/hAβ (1:3000; 6E10, Biolegend), mouse anti‐PSD (1:3000, Cell Signaling Technology), mouse anti‐synaptophysin (1:30,000, ICN Biomedicals), and rabbit anti‐GAP‐43 antibody (1:2000, Santa Cruz Biotechnology). After washing in TBST, blots were incubated with the horseradish peroxidase (HRP)–conjugated secondary anti‐rabbit or anti‐mouse antibody (Santa Cruz Biotechnology) in TBST for 1 h at room temperature (RT). Each blot was re‐probed with rabbit anti‐glyceraldehyde 3‐phosphate dehydrogenase (GAPDH) antibody (1:30,000; Cell Signaling Technology). Signals were quantified densitometrically using iBright Western Blot Imaging System (ThermoFisher Scientific) and expressed as relative values, normalized to the corresponding signals of GAPDH as a loading control. The target protein levels in treated mice were determined relative to the appropriate control value in 5XFAD‐Ctrl mice set to 100%.

### 2.6 Amyloid plaque quantification

For histochemical plaque analysis, thioflavin S stain was used. Ten sections per brain (120 μm apart) were incubated in a 1% aqueous solution of Thioflavin‐S (Sigma, T1892) for 8 min at RT, then for 3 min in 80% and 96% ethanol, rinsed in distilled water, and mounted onto glass slides using a mounting medium with DAPI (Dako). Images were captured on an AxioObserver Microscope Z1 using an AxioVision 4.6 software system (Carl Zeiss), and digital images were exported to ImageJ, version 1.74 (NIH) for quantitative analysis. The maximum entropy threshold was chosen for automatic particle analysis. The total number and the average size of plaques and plaque coverage were determined in the cortical and hippocampal regions at the levels of caudate‐putamen and dorsal hippocampus (approximately +0.74 and −2.06 to bregma, respectively).^27^

### 2.7 Immunohistochemistry (IHC)

Sections were thoroughly rinsed with 0.1 M PBS, pH 7.4 and incubated in 1% glycine solution for 10 min and blocked in 10% normal goat serum in PBS with 0.1% Triton X‐100 for 30 min at RT. To detect microglia, astrocytes, and hAβ, subsequent sections were incubated overnight at 4°C with rabbit anti‐ionized calcium binding adaptor molecule 1 (Iba‐1) (1:1000, Wako Chemicals), mouse anti‐glial fibrillary acidic protein (GFAP) antibody (1:1000, Millipore), and mouse anti‐hAPP/hAβ antibody (1:3000; 6E10, Biolegend), respectively. After rinsing with PBS, sections were incubated with Alexa Fluor 568‐conjugated anti‐mouse secondary antibody (1:500, ThermoFisher Scientific) for 1 h at RT and then rinsed again with PBS. The sections were mounted and coverslipped with a fluorescent mounting medium (Dako).

### 2.8 Quantification of Iba1‐positive microglia, GFAP‐positive astrocytes, and amyloid β positive plaques

Quantification was performed from images taken at 20x magnification on a confocal laser scanning microscope (Leica TCS SP5 II Basic; Leica Microsystems CMS GmbH) with standardized gain, digital offset, and laser intensity. For each animal, layers from 4 to 18 in Z‐stacks for cortical and hippocampal regions were analyzed using ImageJ (NIH), and relative intensity of fluorescence (RIF) of Iba‐1‐, GFAP‐, and hAPP/hAβ‐positive signal was evaluated.

### 2.9 Statistical analysis

All data are shown as scatter plots with single points per mouse and a bar representing the mean ± SEM. Statistical analysis was performed using Statistica 6.0 software (StatSoft Inc.). Normality of data sets was estimated by the Shapiro–Wilk's test. As not all the data sets met the criteria of normal distribution, the differences between the experimental groups were analyzed using non‐parametric Mann–Whitney U‐test. Significance was set at *p* < 0.05.

## 3 RESULTS

### 3.1 The 3HFWC treatment did not affect general health, food and fluid intake, or incite synaptic vulnerability and inflammation in 5xFAD mice

**FIGURE 1:**
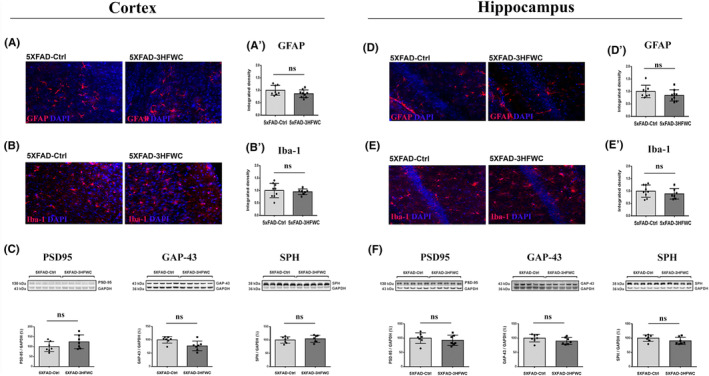
3HFWC treatment did not incite the inflammation or synaptic vulnerability in the cortex and hippocampus of 4‐month‐old 5XFAD female mice. Representative images of GFAP, red, (A, D) and Iba‐1, red, (B, E) staining in the cortex (left) and hippocampus (right) of 5XFAD‐Ctrl and 5XFAD‐3HFWC mice. On all micrographs, DAPI staining is blue. Quantification of GFAP‐positive (A′, D′) and Iba‐1‐positive (B′, E′) staining in the cortex and hippocampus of 5XFAD‐Ctrl and 5XFAD‐3HFWC mice. Scale bar = 25 μm. Immunoblot analysis of PSD‐95 (left plot), GAP‐43 (middle plot), and SPH expression (right plot) in the cortex (C) and hippocampus (F) of 5XFAD‐Ctrl and 5XFAD‐3HFWC mice. Representative immunoblots are shown above the graphs. Relative protein abundances are obtained by normalization relative to GAPDH protein level. Data are shown as mean ± SD (*n* = 7 mice per group). Statistical significance analyzed by one‐way ANOVA.

The 5XFAD‐3HFWC mice did not phenotypically differ from 5XFAD‐Ctrl animals at the end of the treatment. During the treatment, animals were in good health, with no changes in the smoothness and shininess of their fur and home‐cage behavior. There was no difference between 5XFAD and 5XFAD‐3HFWC mice in normalized weight gain (8.36 ± 1.28 and 7.67 ± 1.87 g, respectively) and fluid intake (3.97 ± 0.21 and 4.05 ± 0.32 mL, respectively) However, the studies have shown that the application of carbon‐based nanoparticles can induce an inflammatory response^35^ and affect astrocytes.^36^ Therefore, we assessed the status of astrocytes (GFAP) and microglia (Iba‐1) in the cortex and hippocampus from 3HFWC‐treated 5XFAD mice. Statistical analysis of immunohistochemical staining revealed that the 3HFWC treatment did not induce any changes in GFAP and Iba‐1 expression levels in both brain regions analyzed (Figure 1A,B,D,E).

The application of carbon‐based nanoparticles can be harmful to synaptic plasticity.^37^ Therefore, we assessed the changes in synaptic vulnerability, through the status of synaptic protein markers (PSD‐95, GAP‐43, and SPH), in the cortex and hippocampus of 5XFAD‐ and 5XFAD‐3HFWC‐treated mice (Figure 1C,F). Western blot analysis showed no significant differences in the relative abundances of these proteins in either brain region following 3HFWC treatment.

### 3.2 The detection of 3HFWC treatment induced changes in the 5XFAD mouse brains using machine learning (ML)

In vitro and in silico studies implied that fullerenes can cross biological barriers.^13^, ^14^ In addition, the changes in the fMRI status of specific brain regions induced with 3HFWC treatment suggested the functional effects of on the CNS.^22^ Nevertheless, studies directly verifying that fullerenols cross the blood–brain barrier (BBB) have not been performed so far. To confirm if the 3HFWC treatment induced evident alterations in the CNS, we applied ML through the training of artificial neural networks (ANNs) for the analyses of near infra‐red spectroscopy (NIRS) data.

**FIGURE 2:**
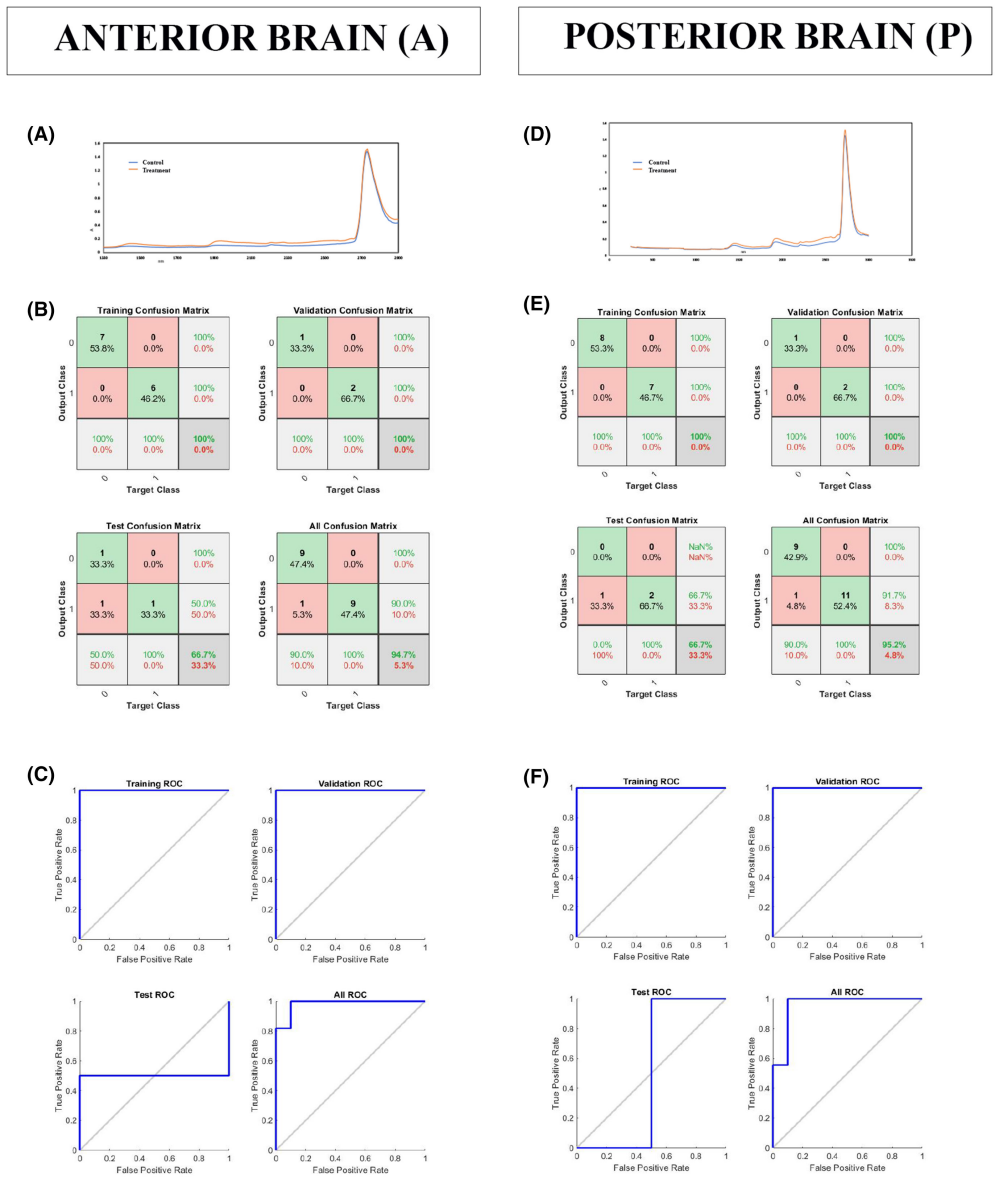
NIRS analyses and confusion matrices. Averaged NIR spectrum for control and treatment samples from anterior (A) and posterior (D) brain (*n* = 10 per group). Confusion matrices from anterior (B) and posterior (E) brain. Green squares represent correct predictions; red ones represent the incorrect ones. The samples from the anterior brain have 94.7% correct prediction and 5.3% incorrect prediction; the samples from the posterior brain have 95.2% correct prediction and 4.8% incorrect prediction. The analyses of ROC curves (colored lines) in the anterior (C) and posterior (F) brain samples showed that they have the value of 0.95 each.

NIRS data contain a huge amount of information, usually of very high dimension, which lends itself to the successful implementation of ML using trained ANN in order to detect pattern differences between control and 3HFWC‐treated brain tissue samples. The ANN performance was evaluated using ROC curves^33^ and AUC score.^34^ The averaged NIR spectra for the control and 3HFWC treated brain samples (*n* = 10 for each group) showed clear differences in the peak value between the control and 3HFWC brain samples in both anterior and posterior brain (Figure 2A,D). The confusion matrices for training, testing, and validation of the ANN performance are shown in Figure 2B,E. ROC curves were created, analyzed, and compared between anterior and posterior brain samples (A and P) (Figure 2C,F and Table 1).

**TABLE 1:** AUC scores from the anterior and posterior brain samples and classifier validation using Recall, Precision, and Accuracy scores.

Anterior	Posterior
AUC scores	
AUC training = 1	AUC training = 1
AUC validation = 1	AUC validation = 1
AUC test = 0.75	AUC test = 0.5
**AUC all = 0.95**	**AUC all = 0.95**
Classifier validation	
Recall = 0.9	Recall = 0.9
Precision = 1	Precision = 1
F measure = 0.94	F measure = 0.94
Accuracy = 0.94	Accuracy = 0.95

Creating a classifier that could classify control and treated brain tissue, the prediction becomes a binary (yes/no) classification problem. In statistical analysis of binary classification, the F‐score (or F‐measure) measures the test's accuracy^38^, ^39^ and determines how comfortable the model is with detecting the positive and negative classes. It is calculated from the precision and recall of the test, where the precision is the number of true positive results divided by the number of all positive results, including those not identified correctly, and the recall is the number of true positive results divided by the number of all samples that should have been identified as positive. The precision scores are presented in Table 1. According to the ROC curve analysis and AUC criterion, the models acquired using anterior brain (A) and posterior brain (P) datasets gave an AUC value of 95% (Table 1).

The values obtained from the analyses of NIRS data using ML suggest that the classifier showed a high accuracy of prediction between the control and 3HFWC‐treated brain samples, confirming that pattern changes in the treated brains are substantial, recognizable, and can be used as a confirmation of the treatment effects.

### 3.3 The 3HFWC treatment decreased the plaque load in the cortex of 5XFAD animals

**FIGURE 3:**
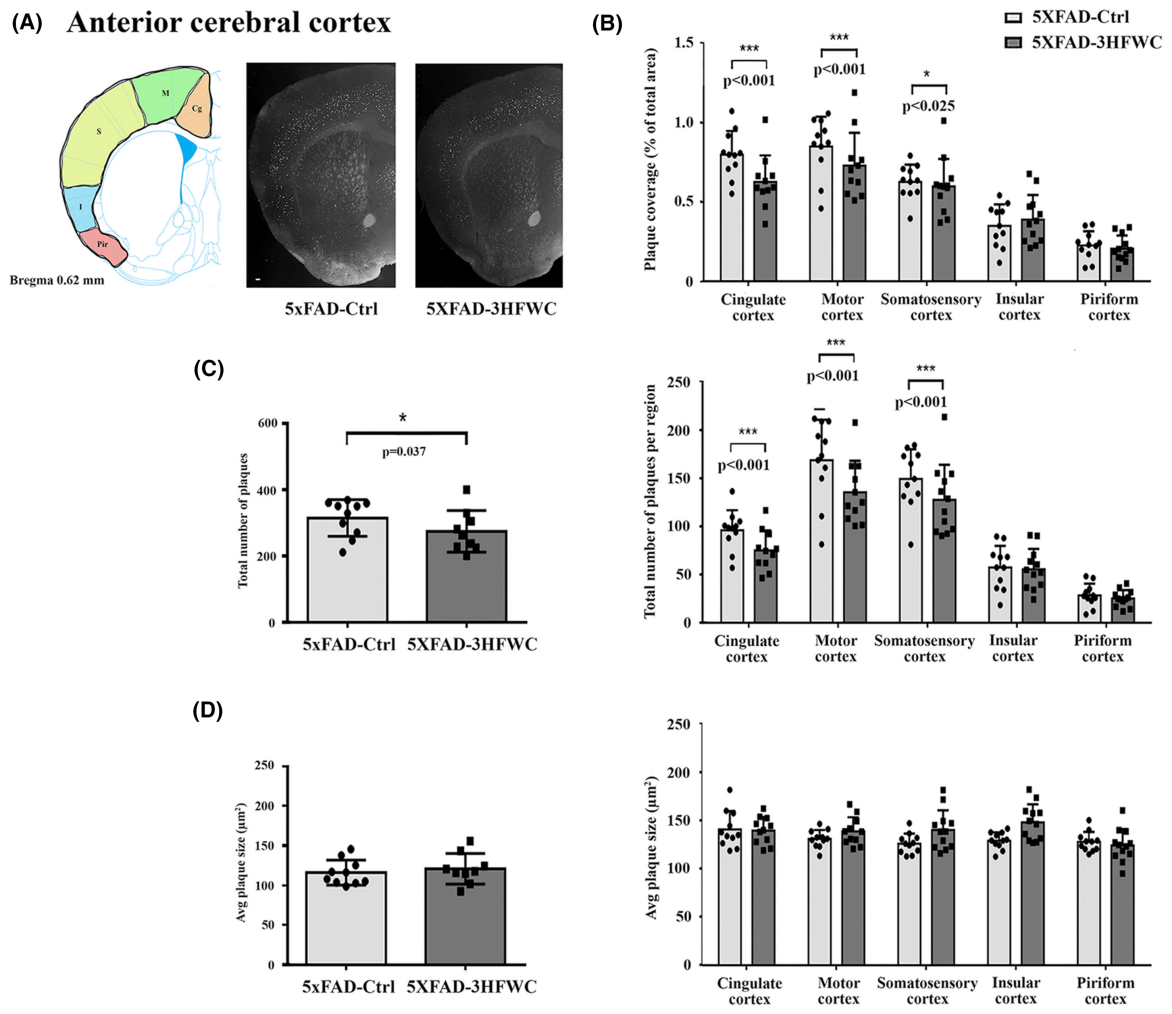
3HFWC treatment reduces the plaque load in the anterior cerebral cortex of 5XFAD female mice. (A) Representative section showing approximate location of cortical regions quantified separately on three serial coronal brain sections (left) and representative photomicrographs of Thioflavin S‐positive amyloid deposits in 4‐month‐old female 5XFAD‐Ctrl and 5XFAD‐3HFWC mice (right). Scale bar = 500 μm. (B) Quantification plot of the percent area covered by Thioflavin S‐positive amyloid plaques in cortical subregions of control (5XFAD‐Ctrl, light gray bars) or mice treated with 3HFWC substance (5XFAD‐3HFWC, dark gray bars). (C) The total plaque numbers in three sections per mouse were counted and are indicated as the number of plaques in the whole cortex (left) or in cortical subregions (right). (D) Average plaque size (μm^2^) in three sections/mouse in the whole cortex (left) or in cortical subregions (right). In all panels, data are shown as mean ± SD (*n* = 10 mice per group). Statistical significance was analyzed by Mann–Whitney U test as the composite (average) histological score from several sections of an individual mouse.

**FIGURE 4:**
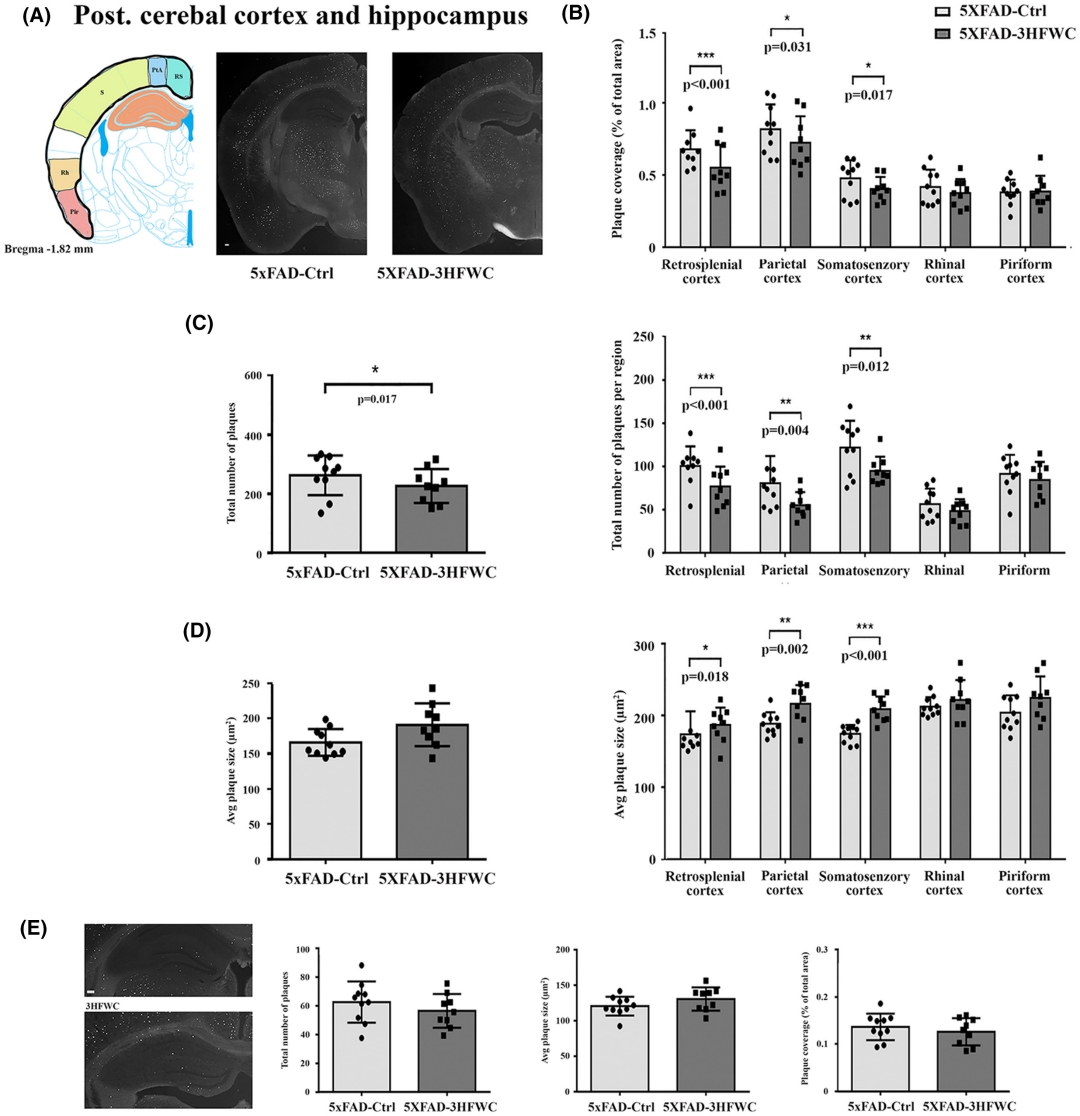
3HFWC treatment reduces the plaque load in the posterior cerebral cortex of 5XFAD female mice. (A) Representative section showing the approximate location of cortical regions quantified separately on three serial coronal brain sections (left) and representative photomicrographs of Thioflavin S‐positive amyloid deposits in 4‐month‐old female 5XFAD‐Ctrl and 5XFAD‐3HFWC mice (right). Scale bar = 500 μm. (B) Quantification plot of the percent area covered by Thioflavin S‐positive amyloid plaques in cortical subregions of control (5XFAD‐Ctrl, light gray bars) or mice treated with 3HFWC substance (5XFAD‐3HFWC, dark gray bars). (C) The total plaque numbers in three sections/mouse were counted and are indicated as the number of plaques in the whole cortex (left) or in cortical subregions (right). (D) Average plaque size (μm^2^) in three sections/mouse in the whole cortex (left) or in cortical subregions (right). (E) Representative photomicrographs of Thioflavin S‐positive amyloid deposits in the hippocampus of 4.5‐month‐old female 5XFAD‐Ctrl and 5XFAD‐3HFWC mice (left) and respective quantification plots. Scale bar = 500 μm. In all panels, data are shown as mean ± SD (*n* = 10 mice per group). Statistical significance analyzed by Mann–Whitney U test as the composite (average) histological score from several sections of an individual mouse.

The fullerene's ability to interfere with Aβ fibril polymerization and aggregation was shown previously.^9^, ^15^, ^16^ Therefore, we examined the 3HFWC treatment effect on the amyloid plaque load and size. ImageJ analysis of Thioflavin‐S‐stained sections revealed a significant decrease in the plaque load following treatment with 3HFWC in the cortex at the levels of putamen and dorsal hippocampus (Figure 3). In comparison to controls, the total plaque burden was reduced in the whole cortex at the level of putamen (Figure 3C, left panel) and dorsal hippocampus (Figure 4C, left panel) (14% and 20%, respectively). Detailed analysis of specific cortical regions revealed that the total plaque number and plaque load was reduced significantly in cingulate (30% and 27%, respectively), motor (28% and 27%, respectively), and somatosensory cortex (18% and 16%, respectively) at the level of the putamen (Figure 3C, right panel). In contrast, the average plaque size was unchanged (Figure 3D). The plaque load and the total number of plaques remained unchanged in the insular and piriform cortex (Figure 3B,C).

A similar reduction in the total plaque number and plaque load after treatment with the 3HFWC compound was observed in the retrosplenial (31% and 32%, respectively), motor (24% and 30%, respectively), and somatosensory (33% and 31%, respectively) cortex of the posterior part of the brain, with no changes in the rhinal, insular, and piriform cortex (Figure 4B). However, the average plaque size was slightly increased in retrosplenial cortex (10%), motor (14%), and somatosensory cortex (19%) (Figure 4D, right panel).

In the hippocampus, neither the total number of plaques, plaque load nor the average plaque size has changed following the treatment with the 3HFWC compound (Figure 4E).

### 3.4 The effect of 3HFWC treatment on hAPP/Aβ load in the cortex of 5XFAD mice

**FIGURE 5:**
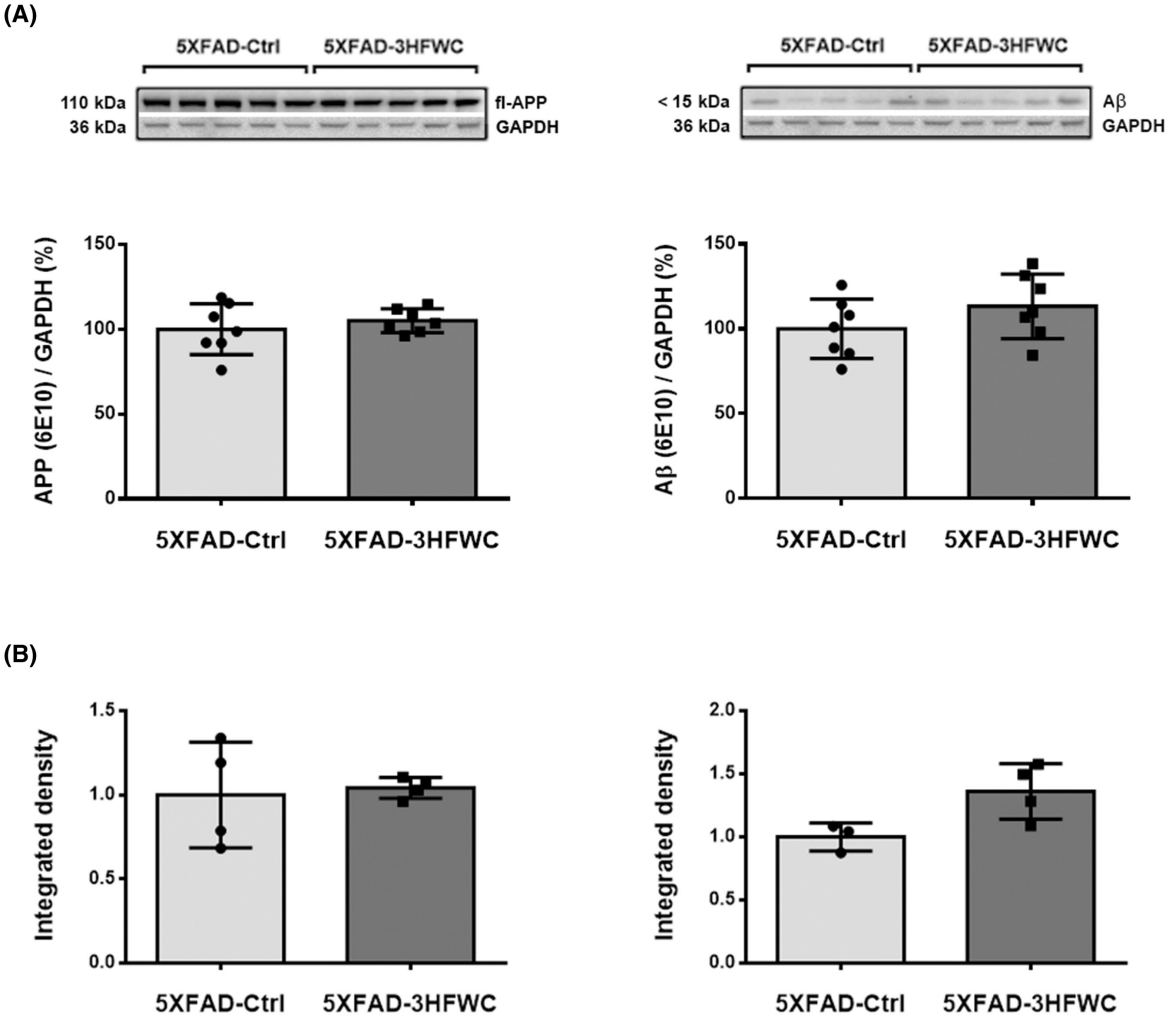
The effect of 3HFWC treatment on APP and Aβ expression in the cortex of 5XFAD mice. (A) Immunoblot analysis of hAPP (left) and hAβ (right plot), detected by 6E10 antibody, in the cortex of 5XFAD‐Ctrl and 5XFAD‐3HFWC mice. Relative protein abundances are obtained by normalization relative to GAPDH protein level. Representative immunoblots are shown above the graphs. (B) Quantification of hAPP/Aβ‐immunostaining in the somatosensory (left) and retrosplenial cortex (right plot) of 5XFAD‐Ctrl and 5XFAD‐3HFWC mice. Data are shown as mean ± SD (*n* = 7 and 4 mice per group for WB and IHH analysis, respectively). Statistical significance analyzed by one‐way ANOVA.

Human APP (hAPP) is overexpressed in transgenic 5XFAD mice. The Western blot analysis using 6E10 antibody detected both hAPP‐ and hAβ‐specific bands (Figure 5A). Semiquantitative analysis of immunoblots revealed that the levels of full‐length human APP and hAβ were the same in both 5XFAD‐Ctrl and 5XFAD‐3HFWC mice, (Figure 5A, left panel). Additional immunohistochemical (IHH) analysis also revealed no change in the relative intensity of fluorescence (RIF) following the staining with 6E10 in the two somatosensory and retrosplenial cortical subregions analyzed (Figure 5B).

## 4 DISCUSSION

The 3‐month‐long 3HFWC treatment, when started in the presymptomatic phase of AD, significantly reduced the plaque load in the specific CNS regions of 5XFAD females without inciting the neuroinflammation and synaptic vulnerability.

The 5XFAD mice represent a valuable AD animal model particularly recommended for studying the effects of different treatments on Aβ deposition.^40^ The 3HFWC treatment in this study was initiated before the onset of pathological hallmarks, as the presymptomatic phase of the disease is considered especially important in assessing the potential of designated therapeutic compounds.^41^ When the first clinical symptoms occur, the disease is so far advanced that current therapeutic agents are not capable of reversing the inflicted damage.

3HFWC is a hyper‐harmonized‐hydroxylated fullerene–water complex and represents a second fullerene derivative. As a third allotropic modification of carbon, fullerene is a hollow, one‐nanometer‐size spheroid structure made entirely of carbon with perfect icosahedral symmetry.^42^ The potential for therapeutic application of fullerenes was further increased by surface functionalization through covalent attachment of hydroxyl groups and the production of fullerenols. The 3HFWC is made by the functionalization of the C_60_ molecule through the addition of OH groups by water layers (wNHS) C_60_(OH)_36 ± 12_@(H2O)_144–2528._ ^21^ These water layers protect surrounding biomolecules from the potentially toxic effects of the C_60_. The application of carbon‐based nanoparticles can be harmful for synaptic plasticity^37^ and induce an inflammatory response.^35^ The lack of elevated gliosis or incited synaptic vulnerability in 3HFWC‐5XFAD mice indicates that the functionalized form of fullerene – 3HFWC did not exert harmful effects on the treated animals, expanding its potential for medical applications.^21^, ^43^, ^44^, ^45^

To estimate the functional effects of 3HFWC on the CNS, we exploited the analytical benefit of NIR spectroscopy for detecting pattern differences between control and 3HFWC‐treated brain tissue samples. Light absorption from the NIR region is primarily caused by functional groups C–H, O–H, and N–H.^46^ When several biomolecules with similar concentrations are present in a matrix, the absorption bands overlap so that at any given wavelength, many substances may contribute to the resulting spectrum.^47^ Using machine learning (ML) through the training of artificial neural networks (ANNs), the highly reliable and accurate AUC values ≥ 0.92 obtained by the chosen classifier revealed the high precision of prediction between the control and treated samples, thus confirming that 3HFWC treatment induced specific changes in the CNS of 5XFAD mice.

The anti‐amyloid properties of fullerene and its derivatives were shown in vitro and in silico.^9^, ^19^, ^48^, ^49^, ^50^ The neuroprotective effect of hydrated fullerene nanoparticles was also shown in the Aβ rat model of AD.^15^, ^16^, ^17^, ^18^ In our study, the oral presymptomatic administration of the 3HFWC nanosubstance in the in vivo transgenic model of AD induced a significant decrease (20%–30%) of plaque burden in specific cortical structures (the somatosensory, parietal, retrosplenial, cingulate, and motor cortex), indicating regional sensitivity to the 3HFWC treatment. The lack of effect of the 3HFWC treatment on the plaque load and size in the hippocampus could be due to the small number of plaques at the time of analyses, making it impossible to obtain measurable differences that can reach statistical significance.

The unaltered expression levels of human APP (hAPP) after the 3HFWC treatment were expected as the hAPP is produced constantly and aggressively under the Thy‐1 promoter in 5XFAD mice. In addition, the levels of Aβ were also unaltered. If the 3HFWC prevents Aβ from further polymerization through binding, as was suggested from the in vitro data,^9^, ^19^, ^48^, ^49^, ^50^ it is possible that such 3HFWC–Aβ complex remains in the brain and is recognized by the anti‐Aβ antibody. It would be important to determine how the rate of 3HFWC clearance affects the distribution of Aβ.

## 5 CONCLUSION

The main benefit of the study is that it provides the first demonstration that 3HFWC treatment has the potential to interfere with AD‐induced plaque load when applied early in the presymptomatic phase of disease development. The extremely aggressive Aβ pathology, in 5xFAD mice with extensive extracellular plaque formation beginning at 2 months of age, is much faster and more hostile compared to the time course of human sporadic AD. In spite of such steep increase in the amyloid deposition, the oral administration of 3HFWC was able to induce a 14%–30% decrease in plaque load, depending on the cortical region analyzed, signifying its beneficial effect. Another benefit of this study is that it confirmed the absence of the increased inflammation, synaptic vulnerability, and gliosis after the 3HFWC treatment, which are the common side‐effects of the treatments with other nano compounds. This study has some limitations. Considering that the response to the treatment varied between the brain regions, it is possible that different 3HFWC concentrations might elicit different outcomes. Thus, one of the limitations of this study is that only one concentration of 3HFWC (0.15 g/L in distilled/tap water 3:1) was tested. Another limitation of the study is that we did not conduct the follow‐up on the anti‐amyloid effects after the treatment was terminated. If these effects persist when 3HFWC nano‐particles are no longer supplied remains to be investigated.

In summary, 3HFWC is potentially a promising AD therapeutic candidate with the added benefit of avoiding common side effects of other compounds. However, further studies on 3HFWC mechanisms of action are warranted to more comprehensively determine the dose and duration of the 3HFWC treatment required to achieve optimal protective effects.

## AUTHOR CONTRIBUTIONS

Conceptualization, MP, SK, and SI; formal analysis, MP, JC, VM, MS, and SI; validation, MP, DjK, and SI; writing – original draft, MP, SK, and SI; writing – review and editing, MP, SK, and SI. All authors have read and agreed to the published version of the manuscript.

## FUNDING INFORMATION

This work was supported by the Ministry of Education, Science and Technological Development of the Republic of Serbia (contracts No.451‐03‐9/2021‐14/200007 and No. 451‐03‐9/2021‐14/200017) and Zepter International foundation (project No. #5/2019).

## CONFLICT OF INTEREST STATEMENT

The authors declare no conflicts of interest.

## Supporting information


Figure S1

## Data Availability

Data supporting the findings of this study are available within the article and in article supplementary material (Figure S1).
