# Supplementary material for: The presymptomatic treatment with 3HFWC nanosubstance decreased plaque load in 5XFAD mouse model of Alzheimer's disease
Source: CNS Neurosci Ther. 2023 Mar 27;30(3):e14188. doi: 10.1111/cns.14188 (PMC10915986; doi:10.1111/cns.14188)
Supplement: Supplementary file 1 — Figure S1 [file CNS-30-e14188-s001.pdf]

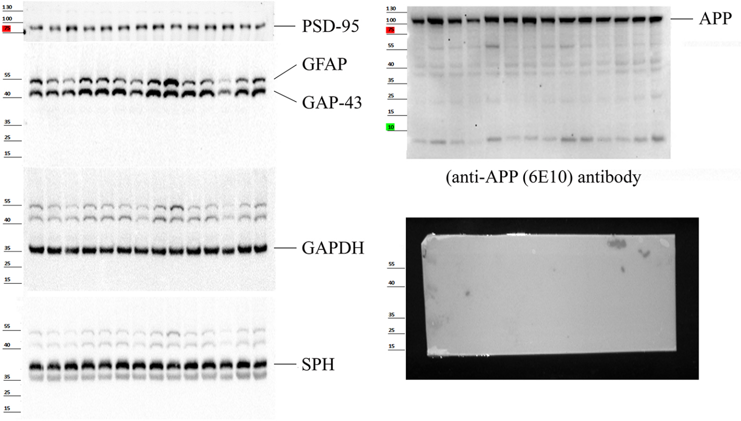

1. Blots cut at 70 kDa
2. > 70 kDa – blotted with anti-PSD-95 antibody (95 kDa)
3. < 70 kDa – blotted with anti-GAP-43 antibody (46 kDa)
4. < 70 kDa – blotted with anti-GFAP antibody (~50 kDa)
5. < 70 kDa – blotted with anti-GAPDH antibody (36 kDa) – end.ctrl
6. < 70 kDa – blotted with anti-SPH antibody (38 kDa)
